# Aspirin to prevent cardiovascular events in patients with community-acquired pneumonia or influenza (ASCAP study): protocol for a multicentre, randomised, double-blind, placebo-controlled trial

**DOI:** 10.1136/bmjopen-2025-110210

**Published:** 2025-11-05

**Authors:** Vahram Hovsepjan, Abel Thijs, Jeske J K van Diemen, Johannes A Bogaards, Michiel M Winter, Judith E Bosmans, Jan M Prins, ASCAP study group, J Altenburg

**Affiliations:** 1 Department of Internal Medicine, Amsterdam UMC, Amsterdam, The Netherlands; 2 Amsterdam Institute for Immunology and Infectious Diseases, The Amsterdam, Netherlands; 3 Amsterdam Cardiovascular Sciences, Amsterdam, The Netherlands; 4 Department of Epidemiology and Data Science, Amsterdam UMC, Amsterdam, The Netherlands; 5 Amsterdam Public Health Research Institute, Amsterdam, The Netherlands; 6 Department of Cardiology, Amsterdam UMC - Locatie AMC, Amsterdam, The Netherlands; 7 Department of Health Sciences, Vrije Universiteit Amsterdam, Amsterdam, The Netherlands

**Keywords:** Respiratory infections, Cardiovascular Disease, INFECTIOUS DISEASES, Myocardial infarction, Randomized Controlled Trial

## Abstract

**Introduction:**

Cardiovascular events (CVEs), in particular acute coronary syndrome (ACS), complicate the course of a significant number of patients hospitalised for community-acquired pneumonia (CAP) or influenza. Emerging evidence suggests that this increased risk of CVEs could be mitigated by the use of acetylsalicylic acid (aspirin). The ASCAP study investigates whether the addition of aspirin to standard therapy in hospitalised patients with moderate-to-severe CAP or influenza can reduce the incidence of CVEs.

**Methods and analysis:**

The ASCAP study is a multicentre, double-blind, placebo-controlled randomised trial in 16 university and general hospitals in the Netherlands, in which patients are randomised to acetylsalicylic acid or matching placebo for 90 days. Eligible patients are adults hospitalised for moderate-to-severe CAP or influenza. Patients with antithrombotic or anticoagulant drugs, or those with contraindications for aspirin, are excluded. The primary outcome is the incidence of ACS up to day 180. Secondary outcomes include the incidence of 4-point major adverse cardiovascular events up to day 180, as well as the incidence of major bleeding and clinically relevant non-major bleeding events up to day 90, all-cause mortality up to day 180 and quality of life and societal costs up to day 180. Survival time will be analysed by the log-rank test, stratified for CAP and influenza, with a two-sided alpha of 0.05. Assuming an average baseline ACS risk of 7.5% over 180 days with up to 30% variation across strata, and a 60% hazard reduction due to aspirin, the required sample size to achieve 80% power is 760 patients. Currently, 114 patients are enrolled in the study.

**Ethics and dissemination:**

This study is approved by the Medical Ethics Committee Amsterdam UMC (Amsterdam, The Netherlands) under reference number 2023.0741 and registered under EU trial number 2023-504553-12-01 in the EU portal CTIS (Clinical Trials Information System). Results of the study will be published in a peer-reviewed journal.

**Trial registration number:**

EU CTIS: 2023-504553-12-01.

**STRENGTHS AND LIMITATIONS OF THIS STUDY**

- Double-blind, randomised, multicentre design with a large sample size.
- Inclusion of patients with moderate-to-severe community-acquired pneumonia, a group at particularly high risk for cardiovascular events.
- Post-intervention follow-up enables assessment of rebound effects and whether aspirin prevents or merely delays cardiovascular events.
- Delays related to the informed consent procedure may lead to the exclusion of the critical early phase following onset of illness.

## Introduction

 Cardiovascular events (CVEs), in particular those confined to the heart, complicate the course of a significant number of patients hospitalised for community-acquired pneumonia (CAP) or influenza.^1^,^3^ For CAP, depending on the definition chosen, the incidence of acute coronary syndrome (ACS) varies between 5% and 8%.^1 4 5^ This increased risk is mostly confined to the first 90 days after the onset of illness, with the highest risk during the first 30 days after hospital admission and gradually declining thereafter.^1 2 6 7^ The incidence is proportional to the severity of the infection, as measured by CURB-65 score or Pneumonia Severity Index (PSI).^1 4 5^

Viral infections, particularly influenza, have also been associated with an increased risk of CVEs. A large UK Biobank study found that the 30-day HR for major CVEs following hospitalisation for a viral infection was 12.75 (95% CI 8.17 to 19.91), after which the risk gradually returned to baseline levels.^2^ Similarly, a self-controlled case series study conducted in the Netherlands showed that the adjusted relative incidence of acute myocardial infarction (AMI) during the risk period (the first week after confirmed influenza) was 6.16 (95% CI 4.11 to 9.24) compared with the control period (1 year before and after influenza).^3^

The occurrence of CVEs, in particular ACS, is associated with a 2.5-fold to 5-fold increase in CAP-associated 30-day mortality.^1 4 5^

There is a growing body of evidence suggesting that this increased risk of CVEs could be mediated, at least partially, by platelets’ response to inflammation (ie, platelet hyperactivity).^8 9^ Because of this hyperaggregability, the question is whether patients with CAP or influenza would benefit from treatment with a platelet inhibitor such as acetylsalicylic acid (aspirin).^10^

There are several observational studies and one randomised controlled trial (RCT) supporting this hypothesis for CAP.^11^,^14^ An observational study prospectively enrolled 1005 patients admitted with community-onset pneumonia and followed them until discharge or death.^11^ Patients already on aspirin therapy experienced fewer intrahospital non-fatal CVEs compared with non-aspirin users (4.9% vs 8.3%). Whereas a PSI V class and severe sepsis or septic shock negatively impacted survival, aspirin therapy was associated with a lower mortality rate (HR 0.43, 95% CI 0.25 to 0.75). A similar—although non-significant—effect of aspirin was noted on 30-day mortality in a prospective observational study of patients with CAP admitted to the hospital.^13^ In a large retrospective cohort study in a primary care setting, aspirin users had a reduced risk of CVEs, including ischaemic stroke and AMI (adjusted HR 0.64, 95% CI 0.52 to 0.79).^12^

The only RCT on this subject enrolled 185 patients with pneumonia who had at least two risk factors for cardiovascular disease.^14^ It was reported that the aspirin group (300 mg once daily) had a significantly lower incidence of ACS at 1 month compared with the control group (1.1% vs 10.6%; relative risk (RR) 0.10; 95% CI 0.005 to 0.746; p=0.015).

While there are no prospective studies specifically examining the effects of prior aspirin use versus non-use in the context of influenza, the use of antithrombotic medication was associated with an almost 70% lower relative incidence of acute myocardial infarction compared with non-use.^3^

Given the mainly observational nature of these studies, a prospective, randomised clinical trial is called for, with the primary objective to investigate whether the addition of aspirin to standard therapy in hospitalised patients with moderate-to-severe CAP or influenza can reduce the incidence of CVEs.

## Methods and analysis

### Study design and setting

The ASCAP study is a multicentre, double-blind, placebo-controlled randomised trial. All administrative information concerning the study is provided in online supplemental appendix A. Participants are randomised in a 1:1 ratio to a 90-day course of acetylsalicylic acid (aspirin) 80 mg or matching placebo, with a total follow-up duration of 180 days. The study is currently being performed in 16 hospitals in the Netherlands (1 university hospital and 15 general hospitals). A list of all study sites can be obtained from the investigators. Inclusion of patients started in January 2024.

### Study population

**Figure 1:**
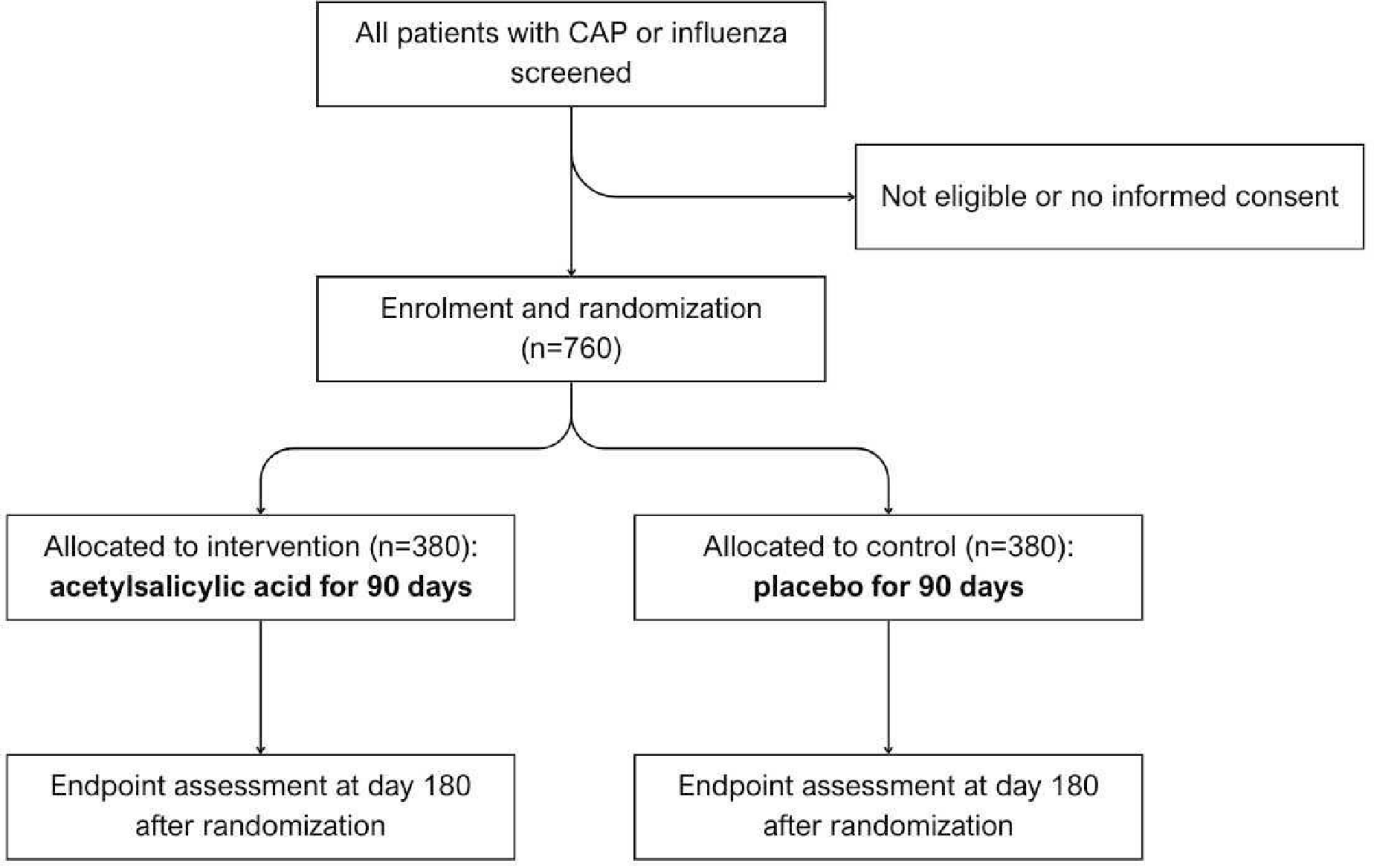
Flow diagram study design ASCAP study. CAP, community-acquired pneumonia.

All patients hospitalised for CAP or influenza will be screened for enrolment in the study. Patients are eligible when they fulfil the inclusion criteria and do not meet any of the exclusion criteria as listed below. A flow diagram for study participants is displayed in figure 1.

### Inclusion criteria

In order to be eligible to participate in this study, a subject must meet the following criteria:

- 40 years or older.
- Any of the following respiratory infections:
  - Moderate-to-severe community-acquired pneumonia, defined as:
    - Clinical signs of pneumonia, and radiological evidence of a new infiltrate consistent with pneumonia, and
    - Severity score: CURB-65 score 2 or higher,^15^ or PSI score 3 or higher,^16^ and
    - No hospitalisation for more than 24 hours in the past 14 days.
  - Influenza: defined as clinical signs of a respiratory infection and a positive influenza PCR test result.
- Admitted to hospital.

### Exclusion criteria

- Conditions which require antiplatelet therapy.
- Use of vitamin K antagonists, direct oral anticoagulants (DOAC) or therapeutic low molecular weight heparin (LMWH).
- Contraindications for aspirin: allergy to salicylates, recent (<1 month) haemorrhagic cerebrovascular accident, recent major gastrointestinal bleeding or other haemorrhage, active bleeding, thrombocytopenia <75×10^9^/L, expected thrombocytopenia <75×10^9^/L (eg, due to chemotherapy in the future), known haemorrhagic diathesis.
- Uncontrolled hypertension (systolic blood pressure >180 mm Hg or diastolic blood pressure >110 mm Hg).
- Life expectancy less than 1 month.
- Pregnancy or breastfeeding.

### Trial intervention

Patients will be randomised to a 90-day course of 80 mg acetylsalicylic acid (with on the first day a loading dose of 160 mg) or matching placebo, with a total follow-up duration of 180 days. Patients have to take the first dose within 84 hours (3.5 days) after hospital admission. Depending on patient characteristics appropriate gastrointestinal bleeding prophylaxis (pantoprazole) will be prescribed during the 90 days that acetylsalicylic acid or placebo is prescribed.^17 18^ Antibiotic treatment, duration of treatment and switch from intravenous to oral antibiotics will be at the discretion of the treating physician, and based on the current Dutch guidelines.^19^

### Trial recruitment, randomisation and blinding

Eligible patients diagnosed with CAP or influenza in the participating hospitals will be approached for written informed consent by the local principal investigator or a designated member of the local study team. However, if the patient is incapacitated due to the acute illness, written informed consent will be obtained from the patient’s representative using an information folder. Once the condition of the patient has improved and the patient information folder can be read, written informed consent will be obtained from the patient. It is important to include these patients to prevent bias in the study population. The model informed consent form can be found in online supplemental appendix B.

Patients are randomised within 84 hours of hospital admission, after all eligibility criteria are verified and met and informed consent has been signed. Participating patients will be entered in an independent centralised database (Castor) with built-in block randomisation to allocate treatment, stratified by centre and relevant prognostic factors with regard to CAP outcome (CURB-65 score: ≤2 or >2) and cardiovascular events (diabetes mellitus: yes or no). Both the patients and the study team will be blinded for treatment allocation.

Randomised patients are replaced and excluded from all further analyses if they have not received the first dose of their study medication or if they have declined to provide written informed consent after initial informed consent by their representative.

### Primary outcome measure

The primary outcome is incidence of an acute coronary syndrome up to day 180 after randomisation, defined using the European Society of Cardiology guidelines.^20^

### Secondary outcome measures

Secondary outcome measures include:

- Incidence of 4-point major adverse cardiovascular events (MACE; composite of non-fatal stroke, non-fatal myocardial infarction, cardiovascular death and coronary revascularisation) up to day 180.
- Incidence of major bleeding complications and clinically relevant non-major bleeding up to day 90.^21 22^
- Length of hospital stay.
- Mortality up to day 180.
- Societal costs up to day 180 measured with iMTA Medical Consumption Questionnaire (IMCQ)^23^ and iMTA Productivity Cost Questionnaire (IPCQ).^24^
- Health-related quality of life measured with the EQ-5D-5L survey.^25^
- Myocardial damage as measured with troponin levels during the first week of hospitalisation.

### Follow-up

At baseline, we will collect a complete medical history and physical examination, C-reactive protein (CRP), white blood cell count and troponin T levels in blood, an ECG, and as per routine clinical care, blood and sputum cultures, PCR on nasal swab for influenza, SARS-CoV2 and respiratory syncytial virus and a urinary pneumococcal and Legionella antigen test. After starting with the trial intervention, ECGs and troponin levels will be measured three times during the first week of hospitalisation. In the subgroup of patients who consent to additional blood sampling and storage for future studies, blood samples will be collected prior to and 4 days after initiating the study medication. We will perform clinical follow-up assessments focusing on cardiovascular complications on day 14, 45, 90 (end of intervention), 135 and 180 (end of study), and an additional ECG will be made on day 90 and 180.

Quality of life will be measured using the EQ-5D-5L questionnaire at four points during the study (at randomisation, day 45, day 90 and day 180). Societal costs will be assessed using structured questionnaires based on the iPCQ and iMCQ. Both will be measured on day 90 and 180. All study procedures are listed in table 1.

**Table 1:** Study procedures ASCAP study

	Study period							
**Days since hospitalisation**	**≤3.5**	**≤3.5**						
**Days since randomisation (±day window**)		**0**	**0–7**	**14** (**±2**)	**45** (**±5**)	**90** (**±5**)	**135** (**±5**)	**180** (**+10**)
Enrolment:								
Eligibility screening	X							
Informed consent	X							
Randomisation		X						
Intervention:								
Acetylsalicylic acid		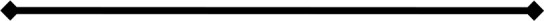						
Placebo		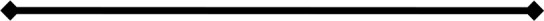						
Pantoprazole if indicated		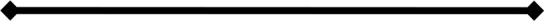						
Assessments:								
Baseline variables*		X						
Troponin		X	X‡					
ECG		X	X‡			X		X
Substudy blood samples†		X	X					
Follow-up				X	X	X	X	X
Adverse events			X	X	X	X	X	X
EQ-5D-5L			X		X	X		X
iPCQ and iMCQ						X		X

* Per routine clinical care: medical history, physical examination, CRP, white blood cell count, blood and sputum cultures, PCR on nasal swab for influenza, SARS-CoV2 and RSV and a urinary pneumococcal and Legionella antigen test.

† On day 0 and 4 after randomisation.

‡ ECG and troponin T three times during the first week of hospitalisation, and on indication.

iPCQ, iMTA Productivity Cost Questionnaire.CRP, C-reactive protein; iMCQ, iMTA Medical Consumption Questionnaire; RSV, respiratory syncytial virus.

### Unblinding

All members of the study team, with the exception of the pharmacy staff, will remain blinded to treatment allocation. In the event of an unblinding request, the study team will assess whether unblinding is necessary for the safety of the patient. Unblinding may only proceed with the explicit permission of the principal investigator. If unblinding is approved, only the clinical care team directly involved with the patient’s treatment will be informed of the allocation (need to know basis). All other individuals involved in the conduct, analysis or interpretation of the clinical trial will remain blinded to ensure the integrity of the study.

### Discontinuation of trial medication

The criteria for temporary or permanent discontinuation of trial medication are detailed below. Participants withdrawn due to medical necessity or meeting exclusion criteria during the course of the study will continue follow-up until day 180, in accordance with the modified intention-to-treat (mITT) analysis plan.

#### Temporary interruption

- Periprocedural setting, if antiplatelet therapy is contraindicated during the procedure.
- Temporary use of antiplatelet therapy, vitamin K antagonists, DOACs or therapeutic LMWH.
- New temporary contraindications for aspirin use, as listed in the exclusion criteria.
- Non-severe bleeding, at the discretion of the treating physician considering the cause and course of the bleeding.
- Inability to take oral medication.

#### Permanent discontinuation

- Patient consent withdrawal.
- Eligibility criteria not met (ie, patient wrongly included).
- New indication for antiplatelet therapy, vitamin K antagonists, DOACs or therapeutic LMWH for the duration of the active treatment phase.
- New permanent contraindications for aspirin use, as listed in the exclusion criteria.
- Severe bleeding, at the discretion of the treating physician considering the cause and course of the bleeding.
- Other urgent medical reasons, at the discretion of the investigator or the treating physician.

### Sample size

The total sample size for the primary outcome is estimated to be 690 patients, based on the stratified log-rank test for time-to-event curves with a two-sided alpha of 0.05 and a power of 80%.^26 27^ This estimation is based on the following assumptions: the average baseline ACS risk is 7.5% over 180 days since hospitalisation, with up to 30% variation between patients with CAP or influenza. This baseline ACS risk is within the 5–8% range reported for ACS incidence over a 1–3 months period among hospitalised patients with CAP.^1 4 5 11^ The assumption of a comparable risk in influenza patients follows from a large study in the UK Biobank and replication cohorts.^2^ The effect of aspirin is assumed to be similar in patients with CAP or influenza. The assumed effect size of 60% reduction in ACS hazard is based on the recently reported HR of 0.46 for aspirin use versus non-aspirin use on myocardial infarction outcomes in a primary care setting,^12^ the RR of 0.10 in an RCT in a high-risk population,^14^ and the 70% reduced incidence of acute myocardial infarction in influenza patients receiving antithrombotic therapy compared with those not receiving such treatment.^3^ Taking into account 5% patients not receiving the allocated intervention and a possible loss to follow-up of 5%, the total number needed to include will be 760 participants.

### Statistical analysis

An adjudication committee, blinded for allocation, will classify the cardiovascular outcomes and the bleeding events using an adjudication charter. Missing data, where applicable, will be imputed with the use of multiple imputation according to the multiple imputation by chained equations algorithm.^28^

All analyses will be performed based on the mITT principle, including all consenting and randomised participants fulfilling the inclusion criteria who have received at least the loading dose of the assigned study medication. In addition, a per-protocol analysis will be performed including only those participants who adhered to the assigned intervention without major protocol deviations. We will perform predefined subgroup analyses for the primary and secondary outcomes with regards to CURB-65 score, diabetes and CAP versus influenza.

#### Baseline characteristics

Descriptive statistics will be used to describe the baseline characteristics. Continuous variables will be reported as means with SD or medians with IQR depending on their distribution. Categorical variables will be described using frequencies and percentages.

#### Primary outcome

The primary analysis will compare the time to first ACS event between the aspirin and placebo groups up to day 180 using Kaplan-Meier survival estimates, with treatment differences assessed using the log-rank test. Patients who died without ACS will be censored at the day of death, and patients lost to follow-up will be censored at the date of last contact. HRs and corresponding 95% CIs will be estimated using Cox proportional hazards models, adjusted for prognostic factors that were considered at randomisation (CURB-65 score and diabetes mellitus), diagnosis (CAP or influenza) and other known risk factors. A sensitivity analysis will be performed using the Fine and Gray competing risk model to estimate ACS-free survival, in which death before an ACS will be considered a competing event.^29^ This is in case of a substantial number of deaths, which might impact our initial analysis.

#### Secondary outcomes

The incidence of the 4-point MACE up to day 180 will be analysed and reported in the same way as the primary outcome, that is, using Kaplan-Meier and Cox proportional hazards analyses. Similarly, a sensitivity analysis will be performed using a competing risk model in which non-cardiovascular death before a 4-point MACE event will be considered a competing event. Incidence of bleeding complications up to day 90 will be compared between the trial groups using cumulative risk estimates. Statistical significance will be evaluated using the χ^2^ test. Length of hospital stay will be compared between treatment groups using a t-test or, in case of skewed data, the Mann-Whitney U test. Mortality up to day 180 will be compared using Kaplan-Meier survival estimates. Differences between the groups will be assessed using the log-rank test. HRs with 95% CI will be calculated using Cox proportional hazards models, adjusted for prognostic and other relevant risk factors. Troponin levels during the first week of hospitalisation will be compared between the treatment groups. We will perform an analysis with repeated measurements over time (random effect model). A longitudinal multilevel model will be used to assess changes in health-related quality of life (EQ-5D-5L) over time between treatment groups, accounting for repeated measures and adjusting for baseline values.

Both a cost-effectiveness analysis (CEA) and a cost-utility analysis (CUA) will be performed from a societal and healthcare perspective, in accordance with the Dutch guidelines for economic evaluations in healthcare.^30^ The CEA will use the primary clinical outcome (ACS at day 180) as the effect measure, while the CUA will use the quality-adjusted life years derived from EQ-5D-5L responses as the effect measure. Incremental cost-effectiveness ratios (ICERs) will be calculated by dividing the difference in mean total costs between the treatment groups by the difference in mean effects between the treatment groups. Uncertainty surrounding the ICERs will be estimated using bias-corrected and accelerated bootstrapping with 5000 replications and presented using cost-effectiveness planes. Cost-effectiveness acceptability curves which show the probability that the intervention is cost-effective compared with placebo at various willingness-to-pay thresholds will also be estimated.

### Potential harms

The main potential harms to consider are the bleeding complications due to aspirin. For that reason, patients already using medication that affects haemostasis, patients with contraindications for salicylates and patients with uncontrolled hypertension will be excluded. Patients at higher risk for gastrointestinal bleeding complications will be identified, and for this group, concomitant pantoprazole will be prescribed during the active treatment phase, according to established Dutch guidelines.^17 18^ Prophylactic use of proton pump inhibitors has been shown to be very effective to prevent upper gastrointestinal bleeding.^31^ Finally, the incidence of major bleeding events will be closely monitored throughout the study. A data safety management board will be established to evaluate the risk-benefit ratio of the study if three or more major bleeding events have occurred after 50% of the (blinded) study group has completed the 90-day treatment period.

### Data collection and management

On enrolment in the study, patients will be assigned a unique study ID based on the specific study site and the order of entry into the study. This will prevent direct linkage of patient data to individual patients. The key to the code will only be stored at the hospital where participants are being treated. Research data are retrieved from the electronic patient records by trained members of the study team, and directly recorded in an electronic case report form using Castor EDC (electronic data capture). The data will be collected and processed in accordance with the General Data Protection Regulation (European Union, EU) 2016/679 (GDPR) and the Dutch Act on implementation of the GDPR (in Dutch: Uitvoeringswet Algemene Verordering Gegevensbescherming). Data will be stored for 25 years after the end of the study, as required by the EU Clinical Trial Regulation 536/2014 (CTR).

Data will be shared with investigators whose proposed use of the data has been approved by the data access committee of the ASCAP study. All individual participant data collected during the trial will be available, after de-identification. To gain access, data requestors will need to sign a data access agreement, on which the data will be available at a third-party website.

The study will be monitored by an independent monitor (quality controller) from the Clinical Monitoring Center of Amsterdam UMC (University Medical Centers) according to the Good Clinical Practice (GCP) guideline for quality of trial conduct and regulatory compliance.

### Patient and public involvement

We have consulted with representatives from Longfonds and Harteraad, two patient foundations dedicated to pulmonary and cardiovascular diseases, respectively. In addition, we have established an advisory board composed of individuals who are either patients with cardiovascular issues themselves or have close contact with patients. During the design and implementation phases of the trial, the advisory board was actively involved in reviewing and providing feedback on study-related materials, including informed consent forms. Throughout the course of the trial, the advisory board will convene at least two times a year to review the study’s progress and offer insights from the patient perspective.

### Ethics and dissemination

This study is approved by the Medical Ethics Committee Amsterdam UMC (Amsterdam, The Netherlands) under reference number 2023.0741 and registered under EU trial number 2023-504553-12-01 in the EU portal CTIS (Clinical Trials Information System). Substantial modifications to the protocol will be submitted to the same committee. For all participating study sites approval to start the study was granted by the board of directors. The study will be conducted in compliance with the Declaration of Helsinki, the ECH E6 GCP guideline, the EU CTR (Regulation No 536/2014) and the Dutch Medical Research Involving Human Subjects Act. A central insurance is taken out for possible harm from trial participation.

The current trial protocol at the time of submission is V.4.0, dated 10 June 2025.

Dissemination to the medical and scientific community will be achieved through publication in peer-reviewed scientific journals and presentations at international scientific conferences. Co-authorship will be based on international guidelines.

## Discussion

There is growing recognition of the increased risk of cardiovascular events in patients with pneumonia and influenza. Several observational studies have reported that prior aspirin use is associated with a reduced incidence of CVEs in these patients. However, it remains unclear whether initiating aspirin therapy after the onset of illness provides similar cardiovascular protection. Additionally, the potential benefit of aspirin in patients without established indications for antiplatelet therapy must be weighed against potential harm. To our knowledge, this study is one of the first RCTs designed to directly investigate these questions.

One significant challenge anticipated in this trial is patient recruitment, particularly given that patients are often incapacitated due to the acute illness. Including severely ill patients is essential, as they have the highest risk for cardiovascular complications. To address this, the study protocol includes the option of obtaining informed consent from a patient’s representative. Once the patient’s condition has improved, written informed consent will be sought directly from the patient.

Key strengths of this study include its double-blind, randomised design, multicentre setting and large sample size, with sufficient statistical power to demonstrate potential benefit of aspirin in hospitalised patients with moderate-to-severe CAP or influenza. A limitation of the study is that the study-related delay of obtaining informed consent and enrolling participants may lead to the exclusion of the critical early phase following onset of illness, during which the risk of CVEs is highest. This may, in turn, reduce the observed efficacy of aspirin as well as the statistical power to demonstrate its benefit.

Overall, the ASCAP study is one of the first clinical trials investigating whether aspirin can reduce the incidence of CVEs in hospitalised patients with CAP or influenza. Given the high annual incidence of these conditions, the study findings have the potential to significantly influence clinical practice. In case of proven efficacy of aspirin, this will improve management strategies resulting in better patient outcomes in these high-risk populations.

## Supplementary material

10.1136/bmjopen-2025-110210 online supplemental file 1

10.1136/bmjopen-2025-110210 online supplemental file 2
