# Supplementary material for: Aspirin to prevent cardiovascular events in patients with community-acquired pneumonia or influenza (ASCAP study): protocol for a multicentre, randomised, double-blind, placebo-controlled trial
Source: BMJ Open. 2025 Nov 5;15(11):e110210. doi: 10.1136/bmjopen-2025-110210 (PMC12593501; doi:10.1136/bmjopen-2025-110210)
Supplement: online supplemental file 1 [file bmjopen-15-11-s001.docx]

**Appendix A: Administrative information**

Trial registration number: 2023-504553-12-01 (EU CTIS)

Protocol version: 4.0

Protocol date: 10 June, 2025

Trial Sponsor: Amsterdam UMC, location AMC, Meibergdreef 9, 1105 AZ Amsterdam, The Netherlands.

Funder: ZonMw, The Netherlands Organisation for Health Research and Development (grant number 10140022110029)

Roles and responsibilities: sponsor and funder: The study sponsor and funder will not have any role in study design; collection, management, analysis, and interpretation of data; writing of the report; and the decision to submit the report for publication. The coordinating centre (Amsterdam UMC) is responsible for general coordination and daily management of the trial.

WHO Trial Registration Data Set

| Data category | Information |
| --- | --- |
| Primary registry and trial identifying number | EU Clinical Trials Information System (euclinicaltrials.eu) 2023-504553-12-01 |
| Date of registration in primary registry | 15 September, 2023 |
| Secondary identifying numbers | n/a |
| Source(s) of monetary or material support | ZonMw, The Netherlands Organisation for Health Research and Development (grant number 10140022110029) |
| Primary sponsor | Amsterdam UMC, Amsterdam, The Netherlands |
| Secondary sponsor(s) | n/a |
| Contact for public queries | Drs. V. Hovsepjan (v.hovsepjan@amsterdamumc.nl) |
| Contact for scientific queries | Drs. V. Hovsepjan (v.hovsepjan@amsterdamumc.nl) |
| Public title | Aspirin to prevent cardiovascular events in patients with pneumonia or influenza (ASCAP) |
| Scientific title | Aspirin to prevent cardiovascular events in patients with community-acquired pneumonia or influenza (ASCAP) |
| Countries of recruitment | The Netherlands |
| Health condition(s) or problem(s) studied | Pneumonia, influenza, cardiovascular events |
| Intervention(s) | Active comparator: acetylsalicylic acid (aspirin) |
|  | Placebo comparator: matching placebo |
| Key inclusion and exclusion criteria | Ages eligible for study: ≥40 years Sexes eligible for study: both Accepts healthy volunteers: no |
|  | Inclusion criteria: patients (≥40 years) hospitalised with moderate to severe community-acquired pneumonia or influenza |
|  | Exclusion criteria: use of anticoagulation drugs, contraindications for aspirin, uncontrolled hypertension, low life expectancy, pregnancy or breastfeeding. |
| Study type | Interventional |
|  | Allocation: randomized intervention model. Parallel assignment. Masking: double blind (subject, caregiver, investigator, outcomes assessor). |
|  | Primary purpose: prevention |
|  | Phase IV |
| Date of first enrolment | 20 February, 2024 |
| Target sample size | 760 |
| Recruitment status | Recruiting |
| Primary outcome(s) | Incidence of acute coronary syndrome up to day 180 |
| Key secondary outcomes | Incidence of 4-points MACE up to day 180, bleeding complications up to day 90, mortality up to day 180, quality of life and societal costs up to day 180 |
