# Supplementary material for: Aspirin to prevent cardiovascular events in patients with community-acquired pneumonia or influenza (ASCAP study): protocol for a multicentre, randomised, double-blind, placebo-controlled trial
Source: BMJ Open. 2025 Nov 5;15(11):e110210. doi: 10.1136/bmjopen-2025-110210 (PMC12593501; doi:10.1136/bmjopen-2025-110210)
Supplement: online supplemental file 2 [file bmjopen-15-11-s002.docx]

**Subject information for participation in medical research**

**Aspirin to prevent cardiovascular events in patients with pneumonia or influenza – ASCAP study**

*Official title (in Dutch): Aspirine ter preventie van cardiovasculaire complicaties bij patiënten met een community-acquired pneumonie of influenza.*

**Introduction**

Dear Sir/Madam,

With this letter, we would like to ask you to take part in a medical study. Participation is voluntary. You have received this letter because you have been admitted to the hospital with a pneumonia or influenza (flu). You can read about the medical study in this information sheet, what it means for you, and what the pros and cons are. It is a lot of information. Can you please read the information and decide if you want to take part? If you want to take part, complete the form in Appendix D.

**Ask your questions**

You can take your decision based on the information in this information sheet. We also suggest that you do this:

- Ask questions to the investigator who gave you this information.

- Talk to your partner, family or friends about this study.

- Ask questions to the independent expert. For contact details, go to appendix A.

- Read the information on [www.rijksoverheid.nl/mensenonderzoek](http://www.rijksoverheid.nl/mensenonderzoek).

1. **General information**

The Amsterdam UMC has set up this study. Investigators (doctors and research nurses) conduct the study in 9 hospitals. It is expected that 760 subjects will take part. The Medical Ethics Review Committee of the Amsterdam UMC has approved the study.

**2. What is the purpose of the study?**

We want to investigate whether aspirin will reduce the incidence of cardiovascular events such as heart attack and stroke in patients with pneumonia or the flu (influenza). To achieve this, we compare the effects of aspirin with those of a placebo. A placebo is a substance or treatment that appears identical to the active drug, but does not contain any active ingredients or have any therapeutic effects.

1. **What is the background of the study?**

It is known that patients who are admitted to the hospital with pneumonia or the flu (influenza) have an increased risk for cardiovascular complications such as a heart attack or stroke. This is not only during hospitalisation, but also in the first period after discharge. Aspirin has proven to be very effective in preventing heart attacks and stroke. For this reason, we want to investigate whether aspirin is also effective in reducing the incidence of these cardiovascular events in patients with pneumonia or the flu.

1. **What happens during the study?**

*How long will the study take?*

Are you taking part in the study? It will take about 6 months in total.

*Step 1: are you eligible to take part?*

You have been diagnosed with pneumonia or the flu, for which you are admitted to the hospital. You are also not yet being treated with blood thinners (anticoagulants). Therefore, you are eligible to participate in this study.

*Step 2: the treatment*

For this study, we have made 2 groups:

- Group 1. The people in this group will receive Aspirin 80 mg once a day
- Group 2. The people in this group will receive placebo once a day (pills that have no active ingredient or therapeutic effect).

If you decide to participate, a draw will decide which treatment you are given. You and the investigator do not know which group you are in. But if it is important for your health, we can look this up. The treatment duration for both regimes is one tablet once a day during 90 days (3 months) with a loading dose of 2 tablets on the first day. If required by guidelines, you will also be prescribed Pantoprazole (used to treat ‘heartburn’) during these 90 days to reduce the small chance of stomach bleeding. In total we will monitor you for the duration of 6 months to investigate long term effects.

*Step 3: study interventions and measurements during hospitalisation*

During hospitalisation, we will draw blood and make an electrocardiogram (ECG) three times a week. We do this to detect a heart attack early. The blood tests are expected to be more frequent than in standard care. Each time one tube of blood (8 ml) will be collected. This amount does not cause any problems in adults. For comparison: if you give blood at the blood bank, you will give 500 ml of blood at a time. Additionally, you will be asked to fill in a questionnaire on the quality of life.

*Step 4: after discharge from the hospital*

After discharge from the hospital, there will be six additional check-ups, of which four will be conducted over the phone and two at the hospital. At 2, 6, 12 and 18 weeks after the start of hospital admission, you will be contacted by phone to inquire about any signs of a heart attack or stroke, medication side effects, and whether you have needed additional medical care. These phone calls will take approximately 5-10 minutes. Around week 12, an additional electrocardiogram (ECG) will be made at the hospital. At 24 weeks, you will have a final check-up at the hospital for the aforementioned questions and an ECG.

We also ask you to complete a questionnaire about your quality of life, your healthcare use, and your work at three different times during the six months. You will receive these questionnaires in weeks 6, 12 and 24. You can fill out these questionnaires online via email or on paper.

Appendix C has a list of the interventions and measurements we carry out during each contact/visit.

*What is the difference with standard care?*

This study is not very different from standard care. The treatment of the pneumonia or the flu will stay the same. Participation will not prolong hospital stay. However, in this study we do require blood tests and ECGs three times a week during hospitalisation. After hospital discharge the standard care usually consists of one appointment in the outpatient clinic. In this study there will be six appointments after discharge, as described above.

1. **Extra storage of blood samples**

In addition to the procedures mentioned above, we would also like to collect additional blood samples. The purpose of this is to conduct research on the possible causes of heart attacks and strokes in patients with pneumonia or the flu. We also want to investigate if certain lab tests can help us determine which patients are at a higher risk for heart attacks and strokes. For this purpose, we would like to collect two blood samples, namely on the day of admission (day 0) and 4 days after admission (day 4). Each time, 10 ml of blood will be collected. These appointments will coincide with the other study appointments.

The collected blood samples will be stored at the Amsterdam UMC for future use within this study. We ask for your consent via the provided consent form. Please see section 11 for further information on the use and storage of your samples.

You may also choose not to participate in this part of the study. This will not affect your participation in the rest of the study.

1. **What agreements do we make with you?**

We want the study to go well. That is why we want to make the following agreements with you:

- You take the medicine in the way the investigator explained to you.
- You do not take ibuprofen during the first three months, because it may reduce the effectiveness of aspirin. However, you may use diclofenac or naproxen.
- During this study you do not take part in any other medical research in relation to blood thinners (anticoagulants) or cardiovascular disease.
- You come to every scheduled appointment.
- You carry the participant card of the study with you. In your wallet, for example. It states that you are taking part in this study. And who to contact if necessary. Show this card when you visit another doctor.
- You should contact the investigator in these situations:
  - You need to start taking other blood thinners (anticoagulants).
  - You are admitted to the hospital for any reason.
  - You no longer want to take part in the study.
  - Your telephone number, address or email address changes.

*Is it OK for you to get pregnant during the study?*

Women who are already pregnant cannot take part in this study because of a higher risk of more blood loss during labour. However, women are allowed to get pregnant during the study, because both aspirin and the placebo tablets are safe to use during pregnancy.

1. **What side effects, adverse effects or discomforts could you experience?**

*Aspirin:*

Aspirin may cause side effects. Some side effects that are common are hypersensitivity reactions in asthma and allergic patients, and stomach complaints. Since aspirin is affecting blood clotting, you may have a higher tendency to bleeding. This can cause hematomas or bleeding in the gastrointestinal tract. However, the likelihood of major bleeding requiring an emergency room visit is low (less than 0.5%). If you have an increased risk for bleeding, the attending physician, following the applicable guidelines, will additionally prescribe pantoprazole (used to treat ‘heartburn’). This is effective in reducing the risk for gastrointestinal bleeding. More information on aspirin can be found in the information leaflet, which you will receive with the medication.

*Placebo:*

In the case of placebo, there are no expected side effects or adverse effects since it does not contain any active ingredients.

*Pantoprazole:*

Pantoprazole can sometimes cause abdominal complaints. More information on pantoprazole can be found in the information leaflet, which you will receive with the medication.

Blood tests:

Taking a blood sample can be a little painful. Or you could get a bruise as a result.

1. **What are the pros and cons if you take part in the study?**

It is important that you weigh the possible advantages and disadvantages carefully before you decide to participate in the study. Your current treatment of the pneumonia or the flu remains unchanged, therefore participating in the study will not affect the course of the disease. With your participation you do contribute to more knowledge about preventing heart attacks. If you are enrolled in the aspirin group, you may benefit from a decreased risk of cardiovascular disease. However, this is not certain, because this is precisely what this research aims to answer.

Cons of participating in the study are the extra time it will cost you and the possible side effects and discomforts. All of these have been described above in sections 4, 6 and 7.

1. **When does the study end?**

In these situations, the study will stop for you:

- All appointments according to the schedule are completed (section 4 and appendix C).
- You suffer a heart attack or stroke.
- You want to stop participating in the study yourself. You can stop at any time. Report this to the investigator immediately. You do not have to explain why you want to stop.
- The investigator thinks it is better for you to stop.
- If you for any reason need to start taking other blood thinners.
- If there is a reason you are not allowed to take aspirin anymore (e.g. in case of major bleeding or low platelets).
- The government or the Medical Ethics Review Committee assessing the study decide that the study should stop.

*What happens if you stop participating in the study?*

If your participation in this study is stopped midway, the appointments will still take place as scheduled. Please inform the investigator if you do not want this. The investigators will use the data and blood samples that have been collected up to the moment that you decide to stop participating in the study. If you wish, we will destroy the collected blood samples. Please let the investigator know.

The entire study ends when all the participants have finished the follow-up.

*Procedures and surgery*

Some procedures require temporary discontinuation of aspirin. In this cases, you may temporarily stop the study medication. Please discuss this with your attending physician and inform the investigator of the study team. There may be emergency situations in which your attending physician wants to know what group (aspirin or placebo) you are in. He/she can contact us for this information.

1. **What happens after the study has ended?**

*Can you continue taking the medicinal products?*

You cannot continue to use the medicinal products you were taking during the study after the study has finished.

*Will you get the results of the study?*

When the study has ended, the investigator will inform you about the most important results of the study. The study is scheduled to end in 2027. The investigator may also tell you what group you were in. Do you prefer not to know? Please tell the investigator. We will not tell you in that case.

1. **What will be done with your data and body material?**

For this study, your data and body material will be collected, used and stored. This includes data such as your name, date of birth, contact information, medical information, and information we collect during the study. For this study, blood samples will be collected for additional lab tests. We collect, use and store your data to answer the questions of this study and to publish the results. We ask your permission to use your data and blood samples.

*How do we protect your privacy?*

To protect your privacy, we give a code to your data and your body material. We only put this code on your data and body material. We keep the key to the code in a safe place in the hospital. When we process your data and body material, we always use only that code. Even in reports and publications about the study, nobody will be able to see that it was about you.

*Who can see your data?*

Some people can see your name and other personal information without a code. This could include data specifically collected for this study, but also data from your medical file. These are people checking whether the investigators are carrying out the study properly and reliably. These persons can access your data:

- Members of the committee that keeps an eye on the safety of the study.
- An auditor who is hired by the investigator.
- National and international supervisory authorities.

These people will keep your information confidential. We ask you to give permission for this access. The Healthcare and Youth Inspectorate can access your personal information without your permission.

*For how long do we store your data and body material?*

We store your data in the hospital for 25 years.

The blood samples collected for the extra part of this study will be stored in the Amsterdam UMC. These samples will be stored for 5 years in order to be able to make new assessments related to this study in the course of this study. If no longer needed, we will destroy your blood samples.

*Can we use your data and body material for other research?*

Your collected data and your stored blood samples may also be important for other medical research in the field of infections and cardiovascular disease. For this purpose, your data will be stored for 25 years and your blood samples for 5 years. The stored blood samples will only be used for new analyses within the scope of this study. Please indicate in the consent form whether you agree with this. Do you not want to give your consent? Then you can still take part in this study. You will get the same treatment.

*What happens if there are coincidental findings?*

It is possible that during the study we discover something that is not directly relevant to the study but is important to your health. In that case, the investigator will contact you and your doctor. You will then discuss what needs to be done with your doctor. The cost of this will fall under your own insurance policy. With the form, you give consent to inform your doctor or specialist.

*Can you withdraw your consent for the use of your data?*

You can withdraw your consent for the use of your data at any time. Please tell the investigator if you wish to do so. This applies both to the use in this study and to the use in other medical research. But please note: if you take back your consent, and the investigators have already collected data for research, they are still allowed to use this information. The investigators will destroy your body material after you withdraw your consent. But if assessments with your body material have been carried out, the investigator can continue to use the results.

*Do you want to know more about your privacy?*

- For general information on your rights when processing personal data, please visit the website of the Dutch Data Protection Authority ([www.autoriteitpersoonsgegevens.nl](http://www.autoriteitpersoonsgegevens.nl)).
- Do you have questions about your rights? Or do you have a complaint about the processing of your personal data? Please contact the person who is responsible for processing your personal data. For this study, this is the Amsterdam UMC, location AMC (see appendix A for contact details and website).
- If you have any complaints about the processing of your personal data, we recommend that you first discuss them with the research team. You can also contact the Data Protection Officer of the Amsterdam UMC. Or you can submit a complaint to the Dutch Data Protection Authority.

*Where can you find more information about the study?*

You can find more information about the study on the website [www.clinicaltrialsregister.eu](http://www.clinicaltrialsregister.eu). After the study ends, the website may show a summary of the results of this study. You can find the study by searching for the number: 2023-504553-12-01.

1. **Will you receive compensation if you participate in the study?**

Participation in this study will not cost you anything. Neither will you get any compensation if you take part in this study. But you will be paid for your extra travel expenses.

1. **Are you insured during the study?**

Insurance has been arranged for everyone who takes part in this study. The insurance pays for damage caused by the study. But not for all damage. You can find more information about this insurance and any exceptions in Appendix B. There it also says to whom you can report damages.

1. **We will inform your doctor, attending specialist and pharmacist.**

The investigator will inform your attending specialist that you are participating in this study. This is for your own safety. If anything about your current diagnosis, medical history or medication use is unclear, we may contact your general practitioner (family doctor), specialist or pharmacist for additional information. You will consent to this via the consent form.

1. **Do you have any questions?**

You can ask questions about the study to the investigator V. Hovsepjan. For independent advice about participating in this study, please contact the independent expert prof. dr. S.E. Geerlings. She knows a lot about the study, but is not a part of this study. If you have complaints about the study, you can discuss them with the investigator or the doctor who is treating you. If you prefer not to do so, you can also contact the complaints officer of the Amsterdam UMC. Appendix A contains all the contact information.

1. **How do you give consent for the study?**

You can first think carefully about this study. Then you tell the investigator if you understand the information and if you want to take part or not. If you want to take part, fill in the consent form that you can find with this information sheet. You and the investigator will both get a signed version of this consent form.

**Thank you for your attention.**

1. **Appendices to this information**

A. Contact details

B. Information about the insurance

C. Schedule of study interventions and measurements

D. Consent form for subject

**Appendix A: contact details for Amsterdam UMC**

Address: Meibergdreef 9, 1105 AZ Amsterdam

Phone number: 020-5669111

**Coordinating investigator:**

V. Hovsepjan

Email: v.hovsepjan@amsterdamumc.nl

**Principal investigator:**

Prof. dr. J.M. Prins

Email: j.m.prins@amsterdamumc.nl

Reachable via the Internal Medicine outpatient clinic

Phone number:

**Independent expert:**

Prof. dr. S.E. Geerlings

Reachable via the Internal Medicine outpatient clinic

Phone number:

**Complaints:**

Complaints officer Amsterdam UMC, location AMC

Email: klachten@amsterdamumc.nl

Phone number:

**Data Protection Officer of the institution:**

More information: www.amsterdamumc.nl/nl/locatie-amc/rechten-en-plichten.htm

Email: privacy@amsterdamumc.nl

**Emergency number:**

In life-threatening situations, call 112.

For other emergency situations related to the investigation, you can contact V. Hovsepjan (phone number).

For more information about your rights, contact the coordinating investigator.

**Appendix B: information about the insurance**

The Amsterdam UMC has arranged insurance for everyone who takes part in the study. The insurance pays for the damage you have suffered because you participated in the study. This concerns damage you suffer during the study or within 4 years after you participated in the study. You must report damage to the insurer within 4 years.

Have you suffered damage as a result of the study? Please report this to this insurer by phone or mail:

The insurer of the study is:

Name insurer: Centramed

Address: Maria Montessorilaan 9, 2719 DB Zoetermeer

Mailing address: Postbus 7374, 2701 AJ Zoetermeer

Telephone number: 070-3017070

Email: schade@centramed.nl

Policy number: 624.528.303

The insurance pays a maximum of *€650,000* per person and *€5,000,000* for the entire study (and *€ 7,500,000* per year for all studies by the same sponsor).

Please note that the insurance does **not** cover the following damage:

- Damage due to a risk about which we have given you information in this sheet. But this does not apply if the risk turned out to be greater than we previously thought. Or if the risk was very unlikely.
- Damage to your health that would also have happened if you had not taken part in the study.
- Damage that happens because you did not follow directions or instructions or did not follow them properly.
- Damage to the health of your children or grandchildren.
- Damage caused by a treatment method that already exists. Or by research into a treatment method that already exists.

These provisions can be found in the 'Besluit verplichte verzekering bij medisch-wetenschappelijk onderzoek met mensen 2015' ('Medical Research (Human Subjects) Compulsory Insurance Decree 2015'). This decision can be found in the Government Law Gazette (<https://wetten.overheid.nl>).

**Appendix C: Overview of study interventions and measurements**

|  | Method of contact | Interventions/measurements |
| --- | --- | --- |
| From hospital admission to discharge | During hospitalisation | - Drawing blood and making an ECG three times a week during the first week.  - Filling in a questionnaire on quality of life.  - Drawing blood upon admission and on day 4 after the start of admission (extra blood samples). |
| 2 weeks after the start of hospitalisation | By phone | - Questions about your health and possible medication side effects. |
| 6 weeks after the start of hospitalisation | By phone | - Questions about your health and possible medication side effects.  - Filling in a questionnaire on quality of life. |
| 12 weeks after the start of hospitalisation | By phone and hospital visit | - Questions about your health and possible medication side effects.  - ECG  - Filling in a questionnaire on quality of life, healthcare use and work |
| 18 weeks after the start of hospitalisation | By phone | - Questions about your health. |
| 24 weeks after the start of hospitalisation | Hospital visit | - Questions about your health.  - ECG  - Filling in a questionnaire on quality of life, healthcare use and work |

**Appendix D: Informed consent form – subject**

Belonging to:

*Aspirin to prevent cardiovascular events in patients with pneumonia or influenza – ASCAP study*

- I have read the information sheet. I was able to ask questions. My questions have been answered well enough. I had enough time to decide if I wanted to take part.
- I know that taking part is voluntary. I also know that at any time I can decide not to take part in the study. Or to stop taking part. I do not have to explain why.
- I give the investigator consent to inform my general practitioner, specialist and/or pharmacist that I am taking part in this study.
- I give consent to request information from my general practitioner, specialist and pharmacist about data for the study if necessary, as stated in the information letter.
- I give consent to give my doctor or specialist information about accidental discoveries made during the study that are important for my health.
- I give consent to collect and use my data and body material. The investigators only do this to answer the questions of this study.
- I know that some people will be able to see all of my data to review the study. These people are mentioned in this information sheet. I give consent to let them see my data for this review.
- I give consent to share my contact information with the investigators at the Amsterdam UMC for the purpose of conducting the telephone interviews and to send the questionnaires.
- Please tick yes or no in the table below.

| I give consent to store my data to use for other research, as stated in the information letter. | Yes ☐ | No ☐ |
| --- | --- | --- |
| I give consent to let me know after the study which treatment I received/in which group I was. | Yes ☐ | No ☐ |
| I give consent to ask me after this study if I want to participate in a follow-up study. | Yes ☐ | No ☐ |

| I want to participate in the part ‘Extra storage of blood samples’:   - I give consent to draw two tubes of 10 ml blood and to store and use this in the study. The blood samples will be stored for this purpose for 5 years. | Yes ☐ | No ☐ |
| --- | --- | --- |

- I want to take part in this study.

My name is : ………………………………..

Email address : ………………………………..

Signature : ……………………………….. Date: ___/___/___

-----------------------------------------------------------------------------------------------------------------

I declare that I have fully informed this subject about the study mentioned.

If any information becomes known during the study that could influence the subject's consent, I will let this subject know in good time.

Investigator name (or their representative): ........................

Signature:……………………… Date: ___/___/___

-----------------------------------------------------------------------------------------------------------------

<if applicable>

Additional information was given by:

Name :………………………………..

Job title :………………………………

Signature :……………………… Date: ___/___/___

-----------------------------------------------------------------------------------------------------------------

*You will receive a complete information sheet, together with a signed version of the consent form.*
