# Supplementary material for: Aspirin to prevent cardiovascular events in patients with community-acquired pneumonia or influenza (ASCAP study): protocol for a multicentre, randomised, double-blind, placebo-controlled trial
Source: BMJ Open. 2025 Nov 5;15(11):e110210. doi: 10.1136/bmjopen-2025-110210 (PMC12593501; doi:10.1136/bmjopen-2025-110210)
Supplement: Supplementary file 3 [file bmjopen-2025-110210.reviewer_comments.pdf]

PEER REVIEW HISTORY

BMJ Open publishes all reviews undertaken for accepted manuscripts. Reviewers are asked to complete a checklist review form and are provided with free text boxes to elaborate on their assessment. These free text comments are reproduced below.

ARTICLE DETAILS

Title (Provisional)

Aspirin to prevent cardiovascular events in patients with community-acquired pneumonia or influenza (ASCAP study): protocol for a multicentre, randomised, double-blind, placebo-controlled trial

Authors

Hovsepjan, Vahram; Thijs, Abel; van Diemen, Jeske J.K.; Bogaards, Johannes A.; Winter, Michiel; Bosmans, J; Prins, Jan M.; ASCAP study group, on behalf of

---

VERSION 1 - REVIEW

**Reviewer**	**1**
**Name**	**Lang, Eddy**
**Affiliation**	**University of Calgary, Emergency Medicine**
**Date**	**28-Sep-2025**
**COI**	

Thank you for the opportunity to review this study protocol and manuscript. It describes an important and ambitious study that will test a plausible hypothesis. There is strong pathophysiologic rationale for what the investigators are proposing. The study is feasible with the research infrastructure and support described in the paper. If completed the study can have important clinical implications for emergency and primary care management of CAP.

VERSION 1 - AUTHOR RESPONSE

Reviewer: 1
Dr. Eddy Lang, University of Calgary
Comments to the Author:
Thank you for the opportunity to review this study protocol and manuscript. It describes an important and ambitious study that will test a plausible hypothesis. There is strong pathophysiologic rationale for what the investigators are proposing. The study is feasible with the research infrastructure and support described in the paper. If completed the study can have important clinical implications for emergency and primary care management of CAP.

Reviewer: 1
If you have selected ‘Yes’ above, please provide details of any competing interests.: Not applicable
